# Mandibular reconstruction using plates prebent to fit rapid prototyping 3-dimensional printing models ameliorates contour deformity

**DOI:** 10.1186/1746-160X-10-45

**Published:** 2014-10-23

**Authors:** Masaki Azuma, Toru Yanagawa, Naomi Ishibashi–Kanno, Fumihiko Uchida, Takaaki Ito, Kenji Yamagata, Shogo Hasegawa, Kaoru Sasaki, Koji Adachi, Katsuhiko Tabuchi, Mitsuru Sekido, Hiroki Bukawa

**Affiliations:** Oral and Maxillofacial Surgery, Graduate school of Comprehensive Human Sciences, Clinical Sciences, University of Tsukuba, Tsukuba, Ibaraki, 305-8575 Japan; Department of Oral and Maxillofacial Surgery, Faculty of Medicine, University of Tsukuba, 1-1-1 Tennodai, Tsukuba, Ibaraki, 305-8575 Japan; Department of Plastic and Reconstruction Surgery, Faculty of Medicine, University of Tsukuba, Tsukuba, Ibaraki, 305-8575 Japan; Department of Neurophysiology, Faculty of Medicine, Shinshu University, Matsumoto, Nagano, 390-8621 Japan

**Keywords:** Medical rapid prototyping, Three-dimensional printing model, Surgical reconstruction, Mandible, Head and neck cancer

## Abstract

**Background:**

Recently, medical rapid prototyping (MRP) models, fabricated with computer-aided design and computer-aided manufacture (CAD/CAM) techniques, have been applied to reconstructive surgery in the treatment of head and neck cancers. Here, we tested the use of preoperatively manufactured reconstruction plates, which were produced using MRP models. The clinical efficacy and esthetic outcome of using these products in mandibular reconstruction was evaluated.

**Methods:**

A series of 28 patients with malignant oral tumors underwent unilateral segmental resection of the mandible and simultaneous mandibular reconstruction. Twelve patients were treated with prebent reconstruction plates that were molded to MRP mandibular models designed with CAD/CAM techniques and fabricated on a combined powder bed and inkjet head three-dimensional printer. The remaining 16 patients were treated using conventional reconstruction methods. The surgical and esthetic outcomes of the two groups were compared by imaging analysis using post-operative panoramic tomography.

**Results:**

The mandibular symmetry in patients receiving the MRP-model-based prebent plates was significantly better than that in patients receiving conventional reconstructive surgery.

**Conclusions:**

Patients with head and neck cancer undergoing reconstructive surgery using a prebent reconstruction plate fabricated according to an MRP mandibular model showed improved mandibular contour compared to patients undergoing conventional mandibular reconstruction. Thus, use of this new technology for mandibular reconstruction results in an improved esthetic outcome with the potential for improved quality of life for patients.

## Background

Patients with head and neck malignancies who undergo life-saving surgery may still suffer from facial deformity. Mandibular reconstruction is a common, but challenging problem in the treatment of malignant head and neck tumors, particularly when mandibulectomy to remove the diseased tissue that results in a very conspicuously altered mandibular contour. Since the mandible has a complex three-dimensional (3D) conformation, it is difficult to reconstruct. Furthermore, any aberration in mandibular structural alignment may lead to functional disturbances due to malocclusion [1]. To solve these problems, computer-assisted simulation and medical rapid prototyping (MRP) models, fabricated with computer-aided design and computer-aided manufacture (CAD/CAM) programs, were recently developed and applied to the reconstructive surgical procedure.

Many researchers have used prebent titanium reconstruction plates molded to MRP models based on CT images, and they report good outcomes from their use in mandibular reconstruction [2–4]. These techniques permit the precise adaptation of the reconstruction plate to the resected mandible, and excellent mandibular symmetry is therefore achieved in considerably shorter operating times. Other methods for generating reconstruction plates have also been utilized. These include the following: 1) the use of 3D CT as a model for bending the reconstruction plates and as a guide for adapting a free fibula bone graft to the resected mandible [5]; 2) the use of reconstruction plates adapted to a stereolithographic 3D printing model [6]; and 3) CAD/CAM model-based use of manufactured custom hydroxyapatite/polyamide scaffolds [7] or titanium mesh trays [8].

Among the above methods, the use of the titanium plates prebent to fit the MRP model has been the most widely adopted, although both advantages and disadvantages of this method have been reported [2, 4, 6, 9, 10]. The primary advantages reported are 1) shorter surgical time, 2) improved adaptation of reconstruction plates, 3) reduced fatigue of the metal, and 4) improved impact on the patient of the disease diagnosis, easier patient education, and a clear method for surgical planning. The reported disadvantages are 1) the requirement of a high-resolution CT scan, 2) cost, and 3) additional steps in surgical planning. Although the advantages clearly outweigh the disadvantages, the results of previous studies have emphasized facial contour and functional recovery, but few reports have quantified and evaluated the clinical outcome in terms of esthetics. In this report, we compared the surgical outcomes of mandibular reconstruction using a conventional method and a method using plates prebent to fit the patient’s MRP model. Here we used postoperative imaging analysis to evaluate MRP-based mandibular reconstruction and found that it resulted in superior mandibular symmetry compared with conventional reconstruction.

## Methods

### Patients

Data were collected retrospectively on consecutive patients referred for oral malignant cancer treatment at the Department of Oral and Maxillofacial Surgery in the Tsukuba University Hospital between January 2007 and December 2013. The clinical characteristics of the patients are presented in Table 1. Twenty-eight patients (18 males and 10 females) requiring hemi-mandibular resection were treated with either MRP model-based prebent reconstruction plates (MRP group, 12 cases) or the conventional method (conventional group, 16 cases). Diagnoses were obtained by biopsy in all cases prior to surgical resection and planned reconstructions. Twenty-seven cases were squamous cell carcinoma and the remaining case was osteosarcoma of the lower alveolus. Informed consent for participation in the study was obtained from participants according to the policy of the Tsukuba University Hospital Ethical Review Board and unlinkable anonymizing data were used for analysis. Approval for this study was obtained from the Tsukuba University Hospital Ethical Review Board (No. H26-44).

**Table 1:** **Clinical characteristics of the patients**

Patient NO.	Age	Sex	Lesion	TNM classification	Pathology	flap	Surgery	Method
1	69	M	rt. Mandibular Gingiva	T4aN0M0	SCC	none	segmental mandibulectomy	Conventional
2	60	M	lt. Mandibular Gingiva	T4aN2bM0	SCC	Fibular free flap	segmental mandibulectomy	Conventional
3	91	M	rt. Mandibular Gingiva	T2N0M0	SCC	none	segmental mandibulectomy	Conventional
4	79	F	rt. Mandibular Gingiva	T2N0M0	SCC	none	segmental mandibulectomy	Conventional
			rt. Maxillary Gingivart. Mandibular Gingiva					
5	42	M	rt. Mandibular Gingiva	T4aN2cM0	SCC	none	segmental mandibulectomy	Conventional
6	66	M	rt. Mandibular Gingiva	T4bN2bM0	SCC	Fibular free flap	segmental mandibulectomy	Conventional
7	70	M	lt. Mandibular Gingiva	rT3N2cM0	SCC	Fibular free flap + Fore arm flap	segmental mandibulectomy	Conventional
8	76	F	lt. Mandibular Gingiva	T2N2bM0	SCC	Fibular free flap	segmental mandibulectomy	Conventional
9	61	M	lt. Mandibular Gingiva	T4aN2cM0	SCC	Fibular free flap	segmental mandibulectomy	Conventional
10	63	M	rt. Mandibular Gingiva	T4aN2bM0	SCC	Fibular free flap	segmental mandibulectomy	Conventional
11	34	F	lt. Mandible	T1N0M0	Osteosarcoma	Rectus abdominis free flap.	segmental mandibulectomy	Conventional
12	81	M	lt. Mandibular Gingiva	T4aN2bM0	SCC	none	segmental mandibulectomy	Conventional
13	52	M	rt. Mandibular Gingiva	T4aN0M0	SCC	none	segmental mandibulectomy	Conventional
14	71	F	rt. Mandibular Gingiva	T4aN2cM0	SCC	none	segmental mandibulectomy	Conventional
15	68	M	Floor of Mouth	rT4aN0M0	SCC	PMMC flap	segmental mandibulectomy	Conventional
16	66	F	rt. Mandibular Gingiva	T4aN0M0	SCC	Fibular free flap	segmental mandibulectomy	Conventional
17	65	M	lt. Mandibular Gingiva	T3N1M0	SCC	Fibular free flap	segmental mandibulectomy	MRP
18	61	M	rt. Mandibular Gingiva	T4aN2bM0	SCC	Fibular free flap	segmental mandibulectomy	MRP
19	59	M	lt. Mandibular Gingiva	T4aN2bM0	SCC	Fibular free flap	segmental mandibulectomy	MRP
20	62	F	rt. Mandibular Gingiva	T4aN0M0	SCC	Fibular free flap	segmental mandibulectomy	MRP
21	59	F	rt. Mandibular Gingiva	T4aN0M0	SCC	Fibular free flap	segmental mandibulectomy	MRP
22	55	M	Floor of Mouth	rT4aN1M0	SCC	Rectus abdominis free flap.	segmental mandibulectomy	MRP
23	62	F	lt. Mandibular Gingiva	T3N0M0	SCC	Fibular free flap	segmental mandibulectomy	MRP
24	75	F	rt. Mandibular Gingiva	T3N0M0	SCC	Fibular free flap	segmental mandibulectomy	MRP
25	58	F	rt. Mandibular Gingiva	T4aN0M0	SCC	Fibular free flap	segmental mandibulectomy	MRP
26	82	M	lt. Mandibular Gingiva	T4aN2cM0	SCC	Rectus abdominis free flap.	segmental mandibulectomy	MRP
27	71	M	lt. Buccal Mucous	T4bN0M0	SCC	Rectus abdominis free flap.	segmental mandibulectomy	MRP
28	62	M	lt. Mandibular Gingiva	T4aN0M0	SCC	Fibular free flap	segmental mandibulectomy	MRP

rTNM: recurrence cases, SCC: squamous cell carcinoma, Conventional: Conventional method, MRP: Method using prebent plate based on medical rapid prototyping model.

### MRP models and surgical planning

**Figure 1:**
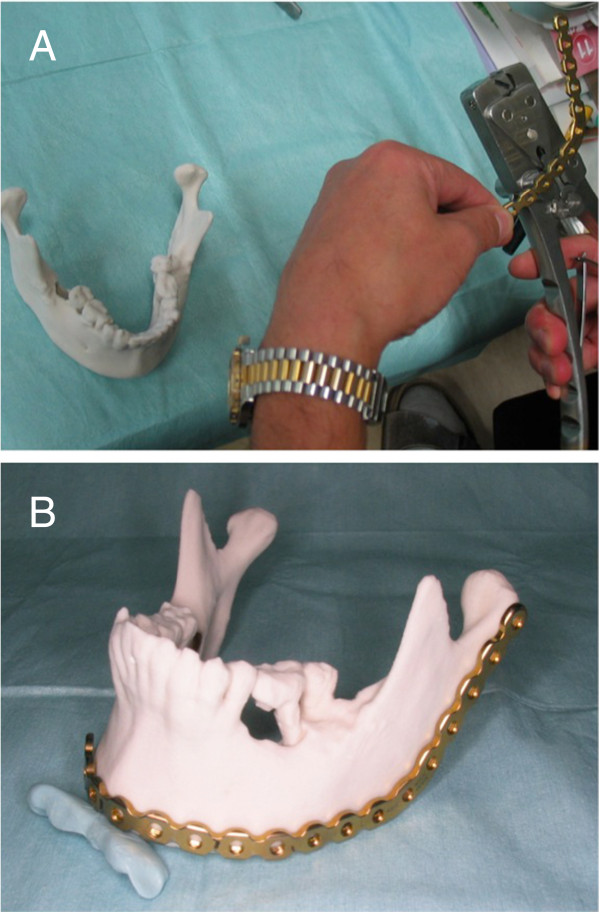
**Manual preparation of the reconstruction plate. A**: Manual preparation of the titanium reconstruction plate adapted to the medical rapid prototyping (MRP) model. **B**: The 3D MRP model with the attached prebent reconstruction plate. Plaster MRP models were obtained using powder bed and inkjet head 3D printing.

High resolution computed tomographic scans (slice thickness less than 2 mm) of the maxillofacial skeleton were obtained and sent to Ahead laboratories Inc, (Tokyo, Japan) for fabrication of the model. Plaster MRP models were obtained using powder bed and inkjet head 3D printing (Zprinter 310+, 3D systems, Rock Hill, USA). The models were sent back to the University Hospital for inspection and surgical planning. Reconstruction margins were designed by the surgeon, and a dental technician in the Department of Oral and Maxillofacial surgery bent and fitted the titanium reconstruction plates (MODUS Reco 2.5, Mediartis, Basel, Switzerland, or Lorenz 2.4 mm locking recon plate, Walter Lorenz Surgical Inc., Jacksonville, USA) to the MRP models (Figure 1A and B). Finished plates were sterilized by autoclaving.

**Figure 2:**
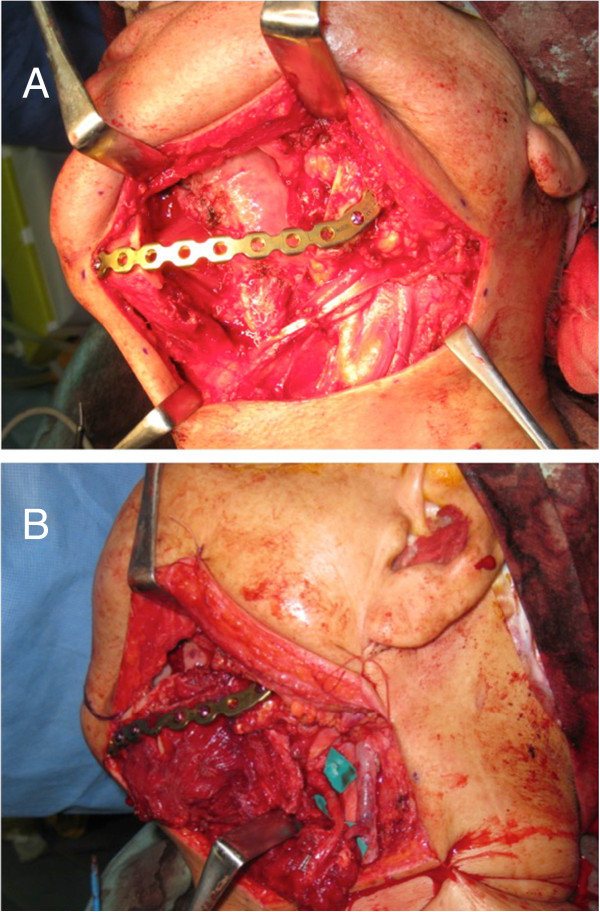
**The reconstructive surgical procedure. A**: Following mandibulectomy, the prebent reconstruction plate based on the MRP model was fixed on residual bone. **B**: The free fibular bone and flap was attached to the inner side of the reconstruction plate.

A two-team approach was used for the surgery. One team consisted of oral and maxillofacial surgeons, who focused on resection of the malignant tumors; the second team consisted of plastic and reconstructive surgeons. An osteotomy was performed to resect the lesion using preset safety margins and intermaxillary fixation, if necessary. Then the prebent reconstruction plate was fixed with titanium screws to the patient’s mandible. Following this procedure, the injured tissue was reconstructed with surgical flaps. A representative case is shown in Figure 2A and B. In this case, the reconstruction plate was fixed to the residual bone, and a free fibular flap was performed, in which fibular bone was adapted to the inner side of the reconstruction plate.

### Surgical outcome evaluation

**Figure 3:**
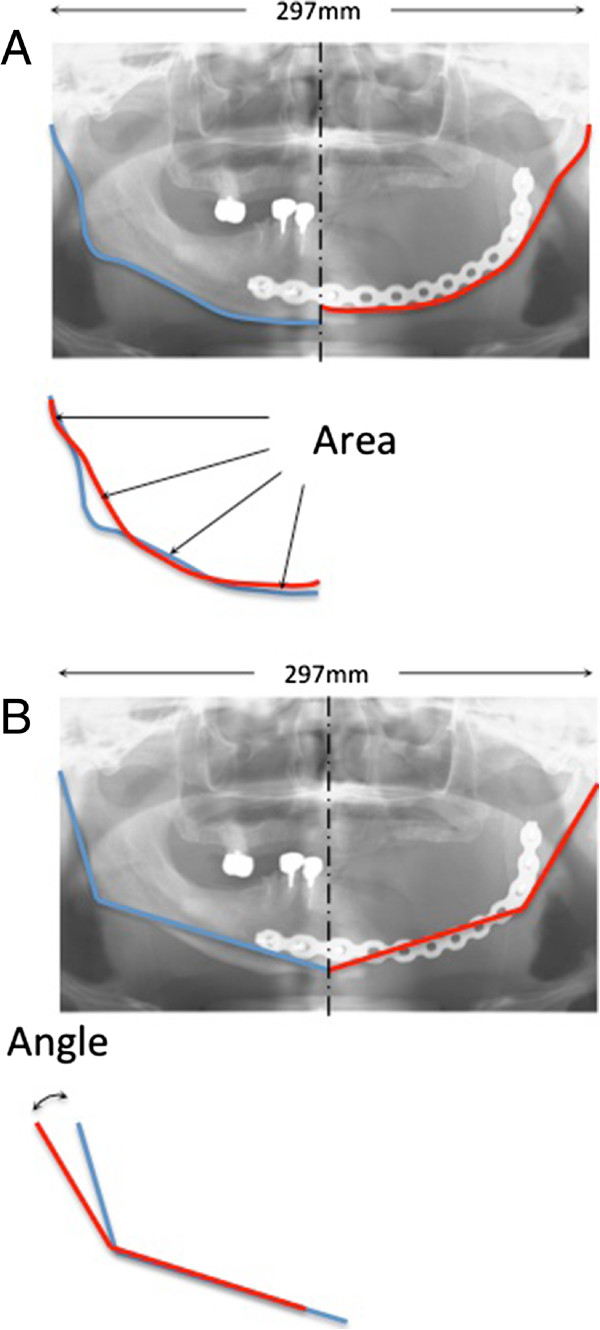
**Symmetry measurement of the reconstructed mandible using imaging analysis. A**: Measurement of the differential area. The mandibular contours from both the reconstructed and unaffected sides were traced, and then the tracings were overlaid. The absolute value of the area contained by the two contour lines was defined as the differential area. **B**: Measurement of the differential angle. The mandibular angles from the unaffected and reconstructed mandibles were measured. The absolute value of the difference between the two angles was defined as the differential angle.

Surgical outcome was determined by evaluating mandibular contour symmetry more than 1 month after surgery. To standardize the different individual mandible sizes, the mandible width (the distance between the most distant point of the mandibular condyle to the contralateral point) was adjusted to 297 mm on the pantomographic film images (AUTO III NR, Asahi Roentgen Industry. Co., Ltd. Kyoto, Japan). First, we traced the mandibular contour on both the reconstructed and unaffected sides. Next, we overlaid the two contour tracings and defined the absolute value of the area contained by the two contour lines as the differential area (Figure 3A). We then defined the absolute value of the difference between the mandibular angles as the differential angle (Figure 3B). Traces were performed twice independently, and the mean values were caluculated (N.I.K). We used Image J 1.45 (National Institute of Mental Health, Bethesda, Maryland, USA) to measure both the differential areas and the angles.

### Statistical analysis

The values of the differential areas and angles in the MRP and conventional groups were analyzed by the Student’s t-test. Differences were assessed with the one-sided test. A P-value <0.05 was considered significant. Statistical analyses were performed with Statcel 3 software (OMS Publisher, Tokorozawa, Japan).

## Results

**Figure 4:**
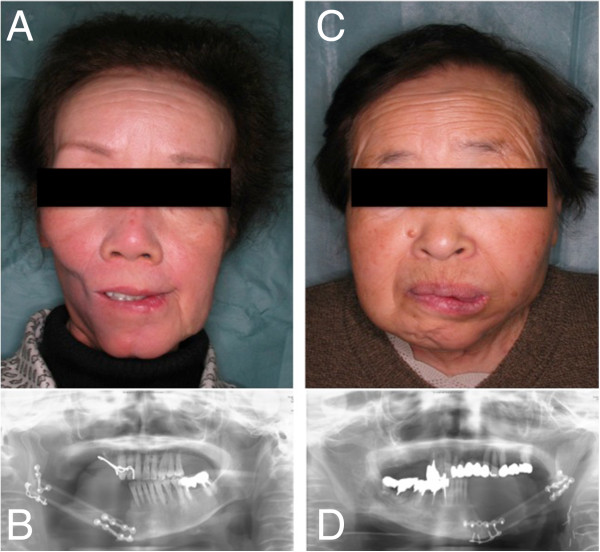
**Representative patients treated with conventional reconstructive surgery. A**, **C**: Images of representative patients following conventional reconstructive surgery using the free fibular flap transfer method. **B**, **D**: Imaging analysis of the above patients using pantomography.

**Figure 5:**
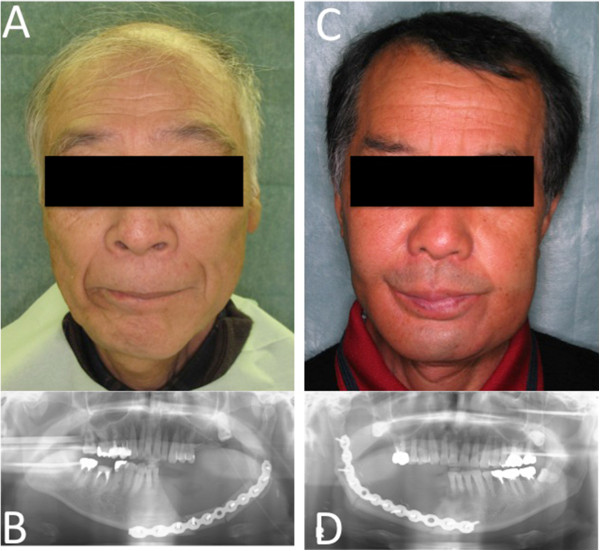
**Images of representative patients following reconstructive surgery using MRP models. A**, **C**: Images of representative patients following reconstructive surgery using the free fibular flap method and prebent reconstruction plates based on MRP models. **B**, **D**: Imaging analysis of the above patients using pantomography.

After resection, the tumor margins were negative for all patients in the study. Representative patients receiving either conventional (Figure 4) or MRP-based (Figure 5) reconstruction are shown for comparison. The two patients shown in Figure 5 clearly exhibited better facial symmetry resulting in an improved esthetic outcome.

**Figure 6:**
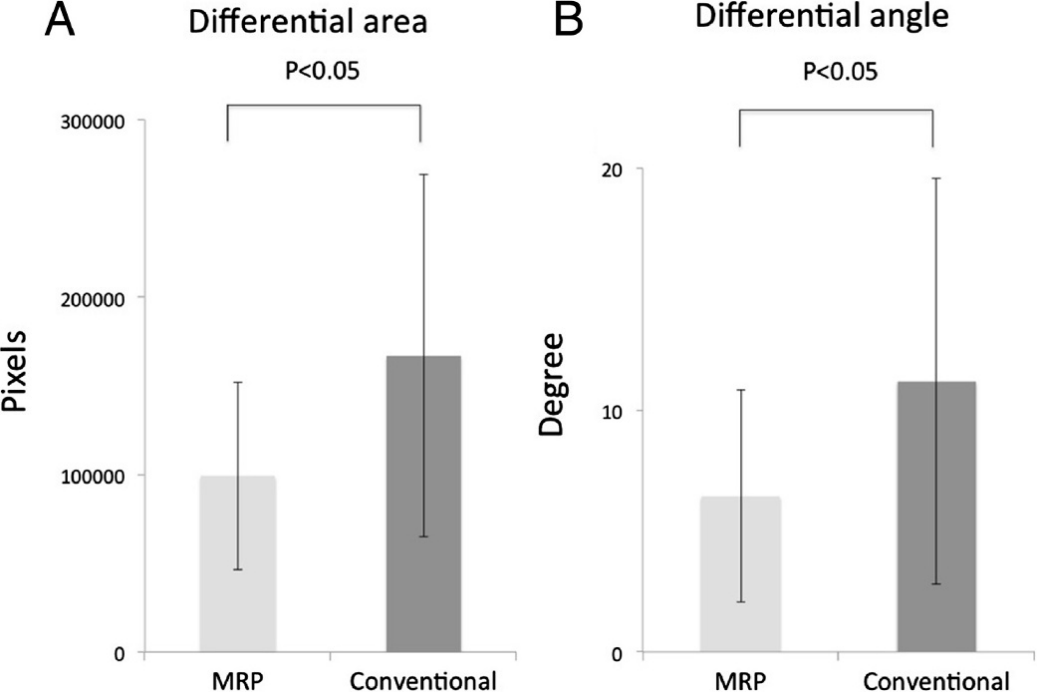
**Imaging analysis results. A**: Chart showing the differential mandibular areas in the two treatment groups measured by imaging analysis. MRP, the group receiving MRP-based reconstructive surgery (MRP group); Conventional, the group receiving conventional reconstructive surgery (Conventional group). Values and error bars indicate the mean ± SD. The differential area of the MRP group was significantly smaller than that of the conventional group (P <0.05). **B**: Chart representing the differential angle of the two treatment groups measured by imaging analysis. Values and error bar indicate the mean ± SD. The differential angle of the MRP group was significantly smaller than that of the conventional group (P <0.05).

Quantitatively, the imaging analysis showed that the differential area in the group receiving MRP model-based reconstruction was 9.92 × 10^4^ ± 5.30 × 10^4^ (mean ± standard deviation, SD) pixels, while that of the group receiving conventional reconstruction was 1.67 × 10^5^ ± 1.02 × 10^5^ pixels (Figure 6). The differential area of the MRP group was significantly smaller than that of the conventional group (P <0.05). Similarly, the differential angle in the group receiving MRP model-based treatment (6.44 ± 4.38 degrees) was significantly smaller than that in the group receiving conventional treatment (11.18 ± 8.39) (P <0.05). These results indicate that the mandibular contour symmetry was significantly improved in the MRP group compared with the conventional group.

## Discussion

In this study we evaluated the esthetic outcome following mandibular reconstruction by symmetry quantification using imaging analysis. The differential mandibular area and angle of the MRP group were significantly smaller than that of the conventional group (P <0.05), indicating that the mandibular contour symmetry resulting from reconstruction using MRP-model-based prebent plates was superior to that obtained with conventional reconstruction methods.

In our method employing MRP models, we fixed the prebent reconstruction plate manually to the residual mandibular bone using the contour line designed presurgically. Recently, various techniques have been developed for the precise transfer of the prebent plate to the appropriate position on the mandible. For example, Klug et al. developed a precise transfer system using stereolithographic models and a computer-assisted navigation tool [11]. In other studies, a splint or mechanical device was developed to transfer the plate to the proper locations [12, 13]. However, malignant tumor resection followed by mandibular reconstruction is complicated by the need to establish a safety margin around the lesion. Therefore, precisely adapting the plate to the mandibular contour during the course of the operation is very difficult.

Furthermore, when mandibulectomy is required to remove a malignant tumor, it can result in the removal of considerable soft tissue, including buccinator and masseter muscle. In these cases, even if the shape of the mandible is successfully restored, the reconstruction may not be esthetically successful, because the soft tissues around the mandible may atrophy after a few months leading to a noticeable contour deformity. In addition, in some cases altered positioning of the masticatory muscle and soft tissues causes mandibular displacement resulting in contour deformity. In this study, we adapted the reconstruction plate to the surface of the inferior border of the mandible (Figure 1A). This approach resulted in a reconstructive plan that was not exactly symmetrical, but the disparity helped to compensate for the soft tissue reduction. Additional studies will be required to improve accurate adaptation of the reconstruction plate to overcome soft tissue problems.

In this study we used pantomography to evaluate mandibular symmetry. Although pantomography is commonly used for evaluating symmetry [14], it is difficult to quantify and compare results across patients with different body sizes. To overcome this problem, we standardized the mandibular images in this study. However, we noticed that the outward appearance of the reconstructed mandibles seemed even better than the imaging results indicated, possibly because the radiograph is a uni-directional image. A bi-directional analysis might clarify this issue and could be helpful in the planning for embedding dental implants into the bone of the defective region, to recover occlusion after surgery. In such cases, axial view analysis will also be necessary for proper evaluation and surgical planning.

The adaptation between the fibula and reconstruction plate is also an important factor. The mandibular contour has a very complex arch; however, the fibula is a linear form, and it is therefore difficult to fit to the curve of the reconstruction plate. In our study, 16 of the 28 cases involved free fibular flap transfer. Of the 9 free fibular flap cases in the MRP group, there was only one case of infection at the reconstruction plate, which was removed 14 months after the reconstructive surgery. Although there were very few such complications, it is possible that the gap between the donor bone and the reconstruction plate affects the infection rate, and this should be taken into consideration. Recently, researchers have obtained 3D fibular images and used them to simulate free fibular flap reconstruction [15–17]. In addition, 3D mandibular and donor site bone images have been used in computer simulations and virtual surgery to optimize the graft’s shape and transfer [15, 18]. Future studies focused on refining fibular flap adaptation to the plate will be beneficial for improving successful clinical outcomes.

Finally, new technological methods are needed to improve the surgical outcomes even more. For example, the development of more effective methods for evaluating 3D information, including soft tissue as well as mandible morphology, will allow more precise analyses, both pre- and post-operatively. Three-dimensional imaging analysis incorporating evaluation methods such as 3D vector analysis [19], Hausdorf distance [20], and moiré topography [21] will permit more precise analysis and lead to improved esthetic outcomes. Moreover, the development of a computerized program that simulates human subjective judgment of the reconstructed facial images could also be beneficial in analyzing esthetic outcomes.

In conclusion, we used MRP models to mold reconstruction plates for mandibular reconstruction following malignant tumor resection and demonstrated that this method resulted in improved esthetic outcomes. Although improved facial esthetics represents an important development in the field of head and neck surgery, there are still some aspects of the method that can be improved. These include: the recovery of soft tissue, the evaluation method, and the precise transfer of the plate and fibula flap. Further improvements in mandibular reconstruction methods and the development of more precise and easier methods for esthetic evaluation will result in better surgical outcomes.

## Conclusions

The use of reconstruction plates that are prebent to fit MRP models in mandibular reconstructive surgery resulted in superior esthetic outcomes compared to the use of conventional reconstructive methods. This is an important development for the field of head and neck surgery and sets the stage for continued improvements in this area.

## Abbreviations

MRP: Medical rapid prototyping
CT: Computed tomography
3D: Three-dimensional
SD: Standard deviation.
